# MRDPDA: A multi‐Laplacian regularized deepFM model for predicting piRNA‐disease associations

**DOI:** 10.1111/jcmm.70046

**Published:** 2024-09-03

**Authors:** Yajun Liu, Fan Zhang, Yulian Ding, Rong Fei, Junhuai Li, Fang‐Xiang Wu

**Affiliations:** ^1^ Shaanxi Key Laboratory for Network Computing and Security Technology, School of Computer Science and Engineering Xi'an University of Technology Xi'an China; ^2^ Center for High Performance Computing Shenzhen Institutes of Advanced Technology, Chinese Academy of Sciences Shenzhen China; ^3^ Department of Computer Science, Biomedical Engineering and Mechanical Engineering University of Saskatchewan Saskatoon Saskatchewan Canada

**Keywords:** DeepFM, Laplacian regularized, piRNA, piRNA‐disease association

## Abstract

PIWI‐interacting RNAs (piRNAs) are a typical class of small non‐coding RNAs, which are essential for gene regulation, genome stability and so on. Accumulating studies have revealed that piRNAs have significant potential as biomarkers and therapeutic targets for a variety of diseases. However current computational methods face the challenge in effectively capturing piRNA‐disease associations (PDAs) from limited data. In this study, we propose a novel method, MRDPDA, for predicting PDAs based on limited data from multiple sources. Specifically, MRDPDA integrates a deep factorization machine (deepFM) model with regularizations derived from multiple yet limited datasets, utilizing separate Laplacians instead of a simple average similarity network. Moreover, a unified objective function to combine embedding loss about similarities is proposed to ensure that the embedding is suitable for the prediction task. In addition, a balanced benchmark dataset based on piRPheno is constructed and a deep autoencoder is applied for creating reliable negative set from the unlabeled dataset. Compared with three latest methods, MRDPDA achieves the best performance on the pirpheno dataset in terms of the five‐fold cross validation test and independent test set, and case studies further demonstrate the effectiveness of MRDPDA.

## 1 INTRODUCTION

Identifying the biomolecule‐disease associations can uncover the pathogenesis of complex disease and thus greatly facilitate the diagnosis, treatment, prognosis and prevention of such diseases.^1^ Biomolecules include proteins, ncRNAs and so on. The current methods for predicting ncRNA‐disease associations mainly focus on miRNA‐disease association (MDA), circRNA‐disease association (CDA) and lncRNA‐disease association (LDA). Methods for predicting piRNA‐disease association (PDA) have gradually attracted researchers' attention.

miRNA is a typical class of ncRNA, which plays an important role in post transcriptional gene expression regulation. In 2007, Zhang et al.^2^ use bioinformatics methods to analyse the relationship between miRNA and cancer, revealing the potential mechanism of miRNA in tumour occurrence and development, which starts the MDA computational research. With the continuous advancement of data accumulation and computing technology, scientists have built specialized databases to collect MDA data and developed various bioinformatics tools to predict MDA. Currently, 53,530 miRNA‐disease association entries which include 1896 miRNA and 2360 diseases are collected in HMDD v4.0.^3^ Huang et al.^4^, ^5^, ^6^ systematically evaluate 29 state‐of‐the‐art MDA prediction models, which utilize data and model fusion. They also speculate the trend about MDA works, such as MDA multiclass classification and so on.

CircRNA and lncRNA are two important kinds of ncRNA, which have attracted widespread attention in gene regulation and disease research. More and more CDAs and LDAs have been identified by biological experiments. In recent 10 years, scientists have proposed a number of computational CDA and LDA models. Wang et al.^7^ introduce 27 CDA models, which are divided into two categories, namely network algorithm‐based and machine learning‐based models. Meanwhile, these two classes of methods are also widely used in LDA studies.^8^ Nowadays, deep learning techniques, particularly graph neural network (GNN), are becoming increasingly prevalent, such as graph auto‐encoder.^9^ In summary, MDA, CDA and LDA studies have become matured after a long period and researchers have started to focus on PDA studies.

piRNAs are the largest and most heterogeneous class of small non‐coding RNAs (sncRNAs) with 24‐32 nt and only discovered in animals.^10^ Different from the above‐mentioned ncRNAs, piRNAs bind to PIWI protein to form RNA‐protein complexes, which are important players in the regulation of various biological processes, involving the silence of transposable elements (TEs),^11^, ^12^ the expression of mRNAs and lncRNAs^13^, ^14^ and so on. In the past decade, growing evidences show that piRNAs are expressed in cancer cells^15^ and regulation of PIWI‐piRNA complexes is related to human complex diseases. Ding et al.^16^ find piR‐823 induces cancer cell stemness in the luminal subtype of breast cancer cells by increasing the expression of gene called DNMTs. Amaar et al.^17^ identify specific piRNAs that play oncogenic (piR‐34871 and piR‐52200) and tumour suppressor (piR‐35127 and piR‐46545) roles in lung cancer. In short, piRNAs are potential biomarkers and drug targets in diagnosis, prognosis and therapeutic efficacy of complex diseases.

So far, many biological experimental techniques have been used to explore associations between piRNA and specific human diseases, like small RNA sequencing and ribosome experiments. Although these experiments can accurately identify genuine PDAs, they are time consuming and laborious. With the development of piRNA studies and accumulation of data, public databases about PDAs have been established, which makes it workable for detecting PDAs by computational methods.

Current computational models for other ncRNAs are not completely applicable to PDA research for two reasons. Firstly, piRNA research started relatively late and PDA data is accumulating which is not as abundant as MDA data. Secondly, the length of piRNA is much shorter than circRNA and lncRNA length, which cause piRNA sequence with low information and weak noise resistance. Consequently, researchers have started to develop the specialized PDA method.

To the best of our knowledge, the first PDA prediction model was proposed in 2020 and since then several machine‐learning algorithms have been developed. Eighteen existing PDA methods (before December 2023) are listed in Table 1, which are grouped into three categories: traditional machine learning methods, GNNs and other deep learning models.

**TABLE 1:** prediction methods of piRNAs‐disease associations.

ID	Name	Description	Dataset	Measurements	A	P	D	Performance	PD	CV	Case study	Ref.	ST
1	iPiDi‐PUL	A method via PU learning based on random forest	piRDisease	PSS, DSS, PGS, DGS	5002	4350	21	AUC = 0.854 ± 0.007	2020.5	5‐CV	AD, head and neck squamous cell carcinoma, breast cancer, gastric cancer	^18^	Web
2	SPRDA (2020)	A matrix completion approach based on the structural perturbation	piRDisease	PSS, PGS, DSS, DGS and gene‐disease association information from DisGeNET	1212	501	22	AUC = 0.9523	2020.7	5‐CV	‐	^19^	‐
3	iPiDA‐sHN	A method via two‐step PU learning strategy and CNN	piRDisease	PSS, DSS, PGS, DGS	5002	4350	21	AUC = 0.8867 ± 0.009	2020.10	5‐CV	AD, head and neck cancer	^30^	‐
4	APDA	A stacked autoencoder and random forest classifier is used	piRDisease	Comprehensive DSS, piRNA sequence descriptor of 3‐mer frequencies	5214	4503	27	AUC = 0.9088 ± 0.0126	2020.10	5‐CV	‐	^31^	‐
5	GAPDA	A method based on graph attention network (GAT)	piRDisease	PGS, DGS, comprehensive DSS, piRNA sequence descriptor of 3‐mer frequencies	1212	501	22	AUC = 0.9038 ± 0.0117	2020.10	5‐CV	‐	^22^	‐
6	DFL‐PiDA	A deep feature learning model based on the convolutional denoising auto‐encoder	piRDisease	DSS, PGS, DGS, piRNA sequence feature similarity	5002	4350	21	AUC = 0.9042	2021.12	5‐CV	‐	^33^	‐
7	iPiDA‐GBNN	A method via gradient‐boosting neural network	piRDisease, ncRPheno, MNDR	PSS, PGS, DGS, disease Jaccard similarity, DSS	8002	5184	33	AUC = 0.9130	2021.12	5‐CV	AD, parkinson Disease	^32^	‐
8	piRDA	A method using two‐step PU learning, CNN and SVM	piRDisease	One‐hot encoding	5002	4350	21	AUC = 0.9500 ± 0.001	2022.2	5‐CV	AD, Cardiovascular diseases, Renal cell carcinoma	^34^	Web
9	iPiDA‐LTR	A method based on information retrieval technology	piRDisease	PSS, DSS	5002	4350	21	AUC = 0.9543	2022.8	5‐CV	Diseases associated with piR‐hsa‐15023 and piR‐hsa‐23210	^20^	Web
10	MSRDA	A method based on multi‐source information and stacked autoencoder	piRDisease	piRNA sequence‐based descriptor, disease semantic‐based descriptor, PGS, DGS	1212	501	22	AUC = 0.9184 ± 0.0015	2022.10	5‐CV	‐	^35^	‐

ID	Name	Description	Dataset	Measurements	A	P	D	Performance	PD	CV	Case study	Ref.	ST
11	GAPDA‐ LGAT	A method based on line graph attention networks (LGAT)	piRDisease	PGS, DGS, piRNA sequence features, disease semantic features	1212	501	22	AUC = 0.9038	2022.10	5‐CV	‐	^23^	code
12	iPiDA‐GCN	A method based on graph convolutional networks (GCNs)	MNDR	PSS, PGS, DGS	11,981	10,149	19	AUC = 0.7149	2022.10	5‐CV	AD, cardiovascular disease, renal cell carcinoma, parkinson disease	^24^	Web, code
13	SPRDA (2022)	A model using the structural perturbation method	piRDisease	PSS, DGS, disease functional similarity	1212	501	22	AUC = 0.9524 ± 0.0085	2022.11	5‐CV	The top 20 scoring disease‐related piRNAs	^21^	Code
14	PDA‐PRGCN	A method based on subgraph projection and residual scaling‐based feature augmentation	MNDR	K‐mer similarity of piRNA sequences and DSS	3446	1478	24	AUC = 0.9464	2023.1	5‐CV	Breast neoplasm, renal cell carcinoma, head and neck neoplasms and AD	^25^	Code
15	PDA‐ GCN	A method based on 2‐layer GCN	MNDR	PSS and DSS	11,981	10,149	19	AUC = 0.7833	2023.4	5‐CV	Breast neoplasms, renal cell carcinoma, head and neck neoplasms and AD	^28^	‐
16	ETGPDA	A method based on the embedding transformation graph convolution network	piRDisease	PSS, PGS, DSS and DGS	5002	4350	21	AUC = 0.9603	2023.5	5‐CV	Head and neck squamous cell carcinoma and AD	^26^	Code
17	iPiDA ‐SWGCN	A method based on supplementary weighted graph convolutional network	MNDR	PSS, DSS	11,981	10,149	19	AUC = 0.8178	2023.6	IT	Renal cell carcinoma, Parkinson's disease and Cardiovascular disease	^27^	Code
18	CLPiDA	A contrastive learning approach	MNDR	PGS, DGS	11,981	10,149	19	AUC = 0.8406	2023.12	5‐CV	AD and lung adenocarcinoma	^29^	Code

Abbreviations: A, associations; AD, Alzheimer's disease; CV, cross validation; D, diseases; DGS, disease GIP kernel similarity; DSS, disease semantic similarity; IT, independent test; P, piRNAs; PD, public date; PGS, piRNA GIP kernel similarity; PSS, piRNA sequence similarity; Ref, refence; ST, service type.

In the early stage of PDA study, traditional machine learning methods were mainly used such as random forest, SVM, logistic regression and so on. iPiDi‐PUL^18^ is a representative method, which is the first PDA predictor by positive unlabeled (PU) learning based on random forest and key features extracted by PCA. Additionally, this group also includes SPRDA (2020),^19^ iPiDA‐LTR^20^ and SPRDA (2022).^21^ These methods are clear in theory, but the performance needs to be further improved and limited web services are available.

Recently, deep learning models with excellent representation learning ability have been widely applied in PDA studies and GNN is the most popular technology. Methods include GAPDA,^22^ GAPDA‐LGAT,^23^ iPiDA‐GCN,^24^ PDA‐PRGCN,^25^ ETGPDA,^26^ iPiDA‐SWGCN^27^, PDA‐GCN ^28^ and CLPiDA.^29^ These studies treat PDA data as dichotomous networks or heterogeneous networks and use GNNs to study the PDA problem as the link prediction task to capture the nonlinear relationship between piRNA and disease. However, some of these methods achieve the good performance based on a tailored PDA dataset where degree of all piRNA nodes is greater than 1. Furthermore, methods based on GNN may cause a memory explosion problem due to the full graph training.

In addition to GNN, other deep learning technologies are used in PDA studies, such as CNN, stacked autoencoder and so on. Methods are iPiDA‐sHN,^30^ APDA,^31^ iPiDA‐GBNN,^32^ DFL‐PiDA,^33^ piRDA^34^ and MSRDA.^35^ Although these methods have made contributions to the discovery of PDAs, the model's generalization ability needs to be strengthened and no code is available.

Besides the limitations in existing PDA methods, the quality and quantity of PDA data should be paid attention. There are only three PDA specific databases (piRDisease 1.0, piRPheno 2.0 and MNDR 3.0) and just two of them (piRDisease 1.0 and MNDR 3.0) are used for constructing PDA dataset. Among them, the datasets based on piRDisease are popular. However, the number of samples in piRDisease are limited and it has not been updated for 5 years. In addition, due to experimental conditions and literature records, there are bias and uncertainty in these databases. The datasets are highly imbalanced and only a very small number of diseases are associated with piRNAs. It is worth noting that there are strong inclusion relations between diseases which do not appear in piRNAs.

In this study, we design a novel method, MRDPDA, for predicting PDAs. Specifically, MRDPDA effectively integrates a deep factorization machine (FM) model with regularizations of several separate Laplacians calculated from multiple yet limited datasets. MRDPDA utilizes three types of piRNA information (piRNAs k‐mer, piRNA local alignment and Gaussian interaction profile (GIP) kernel information) and three types of disease information (diseases semantic information, diseases symptomatic information and GIP kernel information). Instead of simply calculating an average similarity network from these multiple similarities,^36^ MRDPDA regularizes deep FM separately with each individual similarity to obtain the effective embedding features of diseases and piRNAs. Moreover, we propose a unified objective function to combine embedding loss about similarities to ensure that the embedding is suitable for the prediction task. In addition, a relatively balanced benchmark dataset is built from piRPheno, where a deep autoencoder is used to construct a reliable negative set from unlabeled samples. Experiments with the cross‐validation test, independent test and case studies fully demonstrated that MRDPDA outperforms the competing methods for predicting the potential PDAs. The major contributions of this study are enlisted as.

- Propose a novel method (MRDPDA) to predict PDAs, where a deepFM is regularized by multiple Laplacians.
- Construct a relatively balanced benchmark dataset based on piRPheno and build a reliable negative set by a deep autoencoder.
- Demonstrate that MRDPDA can effectively integrate multi‐source yet limited data.

## 2 MATERIALS AND METHODS

### 2.1 Benchmark dataset construction

With the development of PDA studies, three PDA specific databases (piRDisease 1.0, piRPheno 2.0 and MNDR 3.0) with experimental validation have been created. Wei et al.^18^ build the first PDA benchmark dataset based on piRDisease. It contains 5002 experimentally validated PDAs involving 4350 piRNAs but only 21 diseases. Zheng et al.^22^ also construct a dataset from piRDisease, which only focuses on piRNAs with the degree greater than 1 in the PDA network. This dataset only contains 1212 PDAs involving 501 piRNAs and 22 diseases. Hou et al.^24^ collect data from MNDR 3.0 and establish the latest PDA dataset which contains 11,981 PDAs including 10,149 piRNAs but only 19 diseases. piRPheno is a new manually curated database that provides experimentally supported PDA records. We create the first PAD dataset by removing duplicate associations in piRPheno. The resulted dataset contains 4417 experimentally validated PDAs containing 462 piRNAs and 102 diseases. This dataset includes the following two subsets:

(1) $$D_{\mathrm{all}}=D^{P}\cup D^{U}$$

**FIGURE 1:**
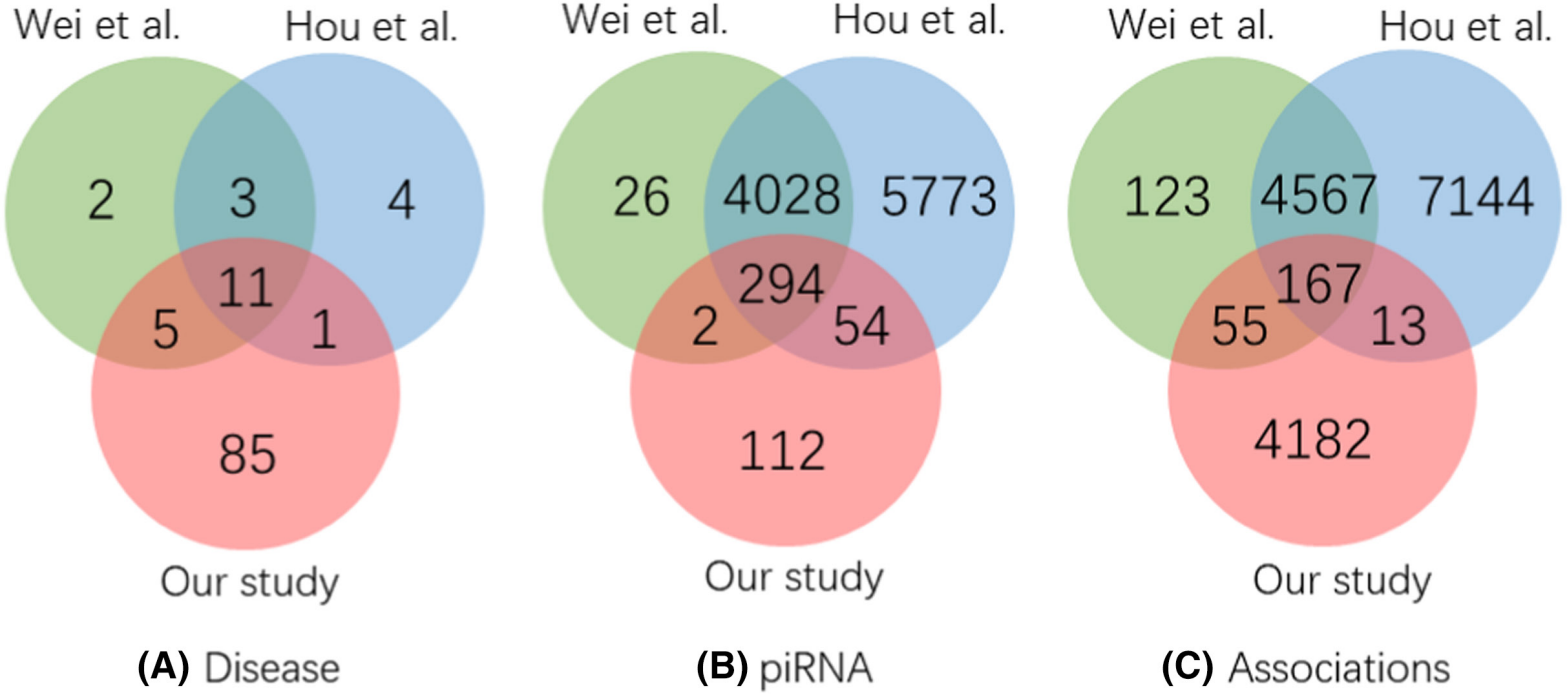
Venn diagram of PDA datasets.

Where $D_{\mathrm{all}}$ is the union of all piRNAs associated among all disease with the total samples, $D^{P}$ is the positive set and $D^{U}$ is the unlabeled set. The relationship of the mentioned three PDA datasets are represented by Venn diagram^37^ as shown in Figure 1.

In our study, PDA data is represented as a PDA matrix A. Specifically, if the i‐th piRNA is experimentally‐validated to be associated with the j‐th disease, the value of $a_{i,j}$ is equal to 1 and 0 otherwise.

### 2.2 Similarity calculation

To obtain both piRNA‐related and disease‐related information from comprehensive views, piRNA‐piRNA and disease‐disease similarities were calculated with various datasets, which are introduced as follows:

#### 2.2.1 piRNA‐piRNA similarity

By mapping name, we collected piRNA sequence from piRBase V3.0 and calculate three piRNA similarities. These similarities are described in the sequel.

- **piRNA sequence local alignment similarity**

The sequence information contains the attribute information of piRNAs and the Smith‐Waterman alignment algorithm can effectively capture the functional similarities among piRNAs. In our study, the piRNA sequence similarity ${\mathrm{PS}}^{1}\left(p_{i},p_{j}\right)$ between the i‐th and j‐th piRNAs is computed as:

(2) $${\mathrm{PS}}^{1}\left(p_{i},p_{j}\right)=\frac{\mathrm{SW}\left(p_{i},p_{j}\right)}{\sqrt{\mathrm{SW}\left(p_{i},p_{i}\right)\times \mathrm{SW}\left(p_{j},p_{j}\right)}}$$

Where the $\mathrm{SW}\left(p_{i},p_{j}\right)$ is the sequence alignment score between the i‐th and j‐th piRNAs, which is calculated by the Smith‐Waterman alignment algorithm.

- **piRNA sequence k‐mer similarity**

The k‐mer term frequency (TF) typically refers to the specific k‐gram frequency of nucleic acid or amino acid sequences which has been widely used to describe the components of biosequences. Inspired by natural language processing methods, we treat a piRNA sequence as a short text and a k‐mer sequence as a word. The word frequency refers to the times of a word which occurs in a corpus of texts. Therefore, we use a sliding window to get a k‐mer frequency vector of one piRNA as a word frequency vector. The cosine similarity is used to measure the piRNA sequence similarity between two vectors and is computed as:

(3) $${\mathrm{PS}}^{2}\left(p_{i},p_{j}\right)=\frac{v_{p_{i}}∙v_{p_{j}}}{\left\|v_{p_{i}}\right\|\times \left\|v_{p_{j}}\right\|\;}$$

Where $v_{p_{i}}$ and$\;v_{p_{j}}$ denote the k‐mer frequency vector of the i th and j th piRNAs.

- **piRNA GIP kernel similarity**

The GIP kernel aims to measure the similarities of biological entities based on their interaction profile information and it has been successfully applied to PDA studies. We compute the GIP kernel similarity as follows:

(4) $${\mathrm{PS}}^{3}\left(p_{i},p_{j}\right)=\exp\left(-\lambda_{p}{\left\|A\left(i,\right)-A\left(j,\right)\right\|}^{2}\right)$$

(5) $$\lambda_{p}={\lambda_{p}}^{\prime }/\left(\frac{1}{m}\sum_{i=1}^{m}{\left\|A\left(i,\right)\right\|}^{2}\right)$$

Where A is the association adjacency matrix between m piRNAs and n diseases, $A\left(i,\right)$ is the i‐th row of A representing the disease‐interaction profile of the i‐th piRNA and the parameter ${\lambda_{p}}^{\prime }$ for controlling kernel bandwidth is set as 1.

#### 2.2.2 Disease‐disease similarity

By collecting disease information from MeSH and Human symptoms‐disease network, we calculate three disease similarities, which are described in sequel.

- **Disease symptom similarity**

The disease symptom similarity is downloaded from Human symptoms‐disease network data in Zhou et al.^38^ Here, the weight ${\mathrm{DS}}^{1}\left(d_{i},d_{j}\right)$ between the i‐th disease and j‐th disease quantifies the similarity of their respective symptoms.

- **Disease semantic similarity**

Disease semantic similarity has successfully been used in various ncRNA‐disease association prediction studies. Using MeSH information, a directed acyclic graph (DAG) is constructed based on hierarchical descriptors, in which nodes represent disease terms and the edges represent the relationship between the current node and its ancestors. ${\mathrm{DAG}}_{d_{i}}=\left(T_{d_{i}},E_{d_{i}}\right)$ describes the structure of the i‐th disease $d_{i}$, where $T_{d_{i}}$ indicates the set of nodes including all ancestors of $d_{i}$ as well as itself and $E_{d_{i}}$ stands for the corresponding direct edges. $D_{d_{i}}\left(t\right)$ denotes the semantic contribution of disease term $t\in T_{d_{i}}$ related to the i‐th disease and can be computed as follows:

(6) $$D_{d_{i}}\left(t\right)=\left\{\begin{array}{c} 1\;\text{if}\;t=i\;\mathrm{th}\;\text{disease}\\ \max\left\{∆\times D_{d_{i}}\left(t^{\prime }\right)|\;t^{\prime }\in \text{children of}\;t\right\}\;\text{otherwise} \end{array}\right.$$

where ∆ is the semantic contribution factor (∆ = 0.5 is the default in this study).

The disease semantic similarity can be defined as follows:

(7) $${\mathrm{DS}}^{2}\left(d_{i},d_{j}\right)=\frac{\sum_{t\in T_{d_{i}}\cap T_{d_{j}}}\left(D_{d_{i}}\left(t\right)+D_{d_{j}}\left(t\right)\right)}{\sum_{t\in T_{d_{i}}}D_{d_{i}}\left(t\right)+\sum_{t\in T_{d_{j}}}D_{d_{j}}\left(t\right)}$$

- **Disease GIP kernel similarity**

The disease GIP kernel is computed by the same way as piRNA GIP kernel similarity as follows:

(8) $${\mathrm{DS}}^{3}\left(d_{i},d_{j}\right)=\exp\left(-\lambda_{d}{\left\|A\left(,i\right)-A\left(,j\right)\right\|}^{2}\right)$$

(9) $$\lambda_{d}={\lambda_{d}}^{\prime }/\left(\frac{1}{m}\sum_{i=1}^{m}{\left\|A\left(,i\right)\right\|}^{2}\right)$$

Where $A\left(,i\right)$ is the i‐th column of A representing the piRNA‐interaction profile of the i‐th disease. The parameter ${\lambda_{d}}^{\prime }$ for controlling kernel bandwidth is set as 1 too.

### 2.3 Selection of reliable negative samples

If negative samples are randomly selected from unlabeled samples, noise may be caused by the fact that not are all unlabeled samples negative samples. To building an accurate model, reliable negative samples are chosen with a deep autoencoder‐based negative sample selection model in this study.

First, each row of PDA matrix A is used as the piRNA feature representation, while each column of matrix A is intended for the disease feature representation. A deep autoencoder is trained by learning the latent features of all PDA samples from $D^{P}$. The i‐th piRNA‐disease sample is defined as $x_{i}=\left[D_{d},P_{p}\right]\in R^{\left(m+n\right)}$, where $D_{d}$ is feature representation of the disease in $x_{i}$ and $P_{p}$ is the feature representation of the piRNA in $x_{i}$. Using $x_{i}$ as input, the encoder extracts the features in a low‐dimensional latent space $z_{i}$ and the decoder reconstructs the input $x_{i}$ based on the latent representation $z_{i}$ from the encoder. The loss of the autoencoder is the average of the reconstruction errors of all the training samples, which describes as follows:

(10) $$L\left(x_{i},x_{i}^{\prime }\right)=-\frac{1}{K\left(m+n\right)}\sum_{i=1}^{K}\sum_{j=1}^{m+n}\left(x_{\mathrm{ij}}\log\left(x_{\mathrm{ij}}^{\prime }\right)+\left(1-x_{\mathrm{ij}}\right)\log\left({1-x}_{\mathrm{ij}}^{\prime }\right)\right)$$

Where $x_{i}^{\prime }$ is the reconstruction of input $x_{i}$, K is the total number of PDA samples in $D^{P}$, m+n is the length of the representation vector of piRNA‐disease pair, $x_{\mathrm{ij}}$ is the j‐th factor of vector $x_{i}$. All the parameters in the autoencoder are updated iteratively by minimizing the loss above.

Finally, all the unknown PDAs are input into the well‐trained model and the reconstruction error is calculated for each unknown piRNA‐disease pair. The reconstruction error scores are sorted in descending order and the unlabeled samples divided into three clusters of nearly the same size according to the scores. The second cluster of unlabeled samples are considered as samples with minimum chance to the false negative. To keep the balance of negative and positive training samples, samples randomly selected from the second cluster with the same size as positive set are judged as reliable negative samples.

### 2.4 Prediction model construction

DeepFM is an end‐to‐end learning frame combining FM and Multi‐Layer Perceptron (MLP), which has the strong capability of automatic learning low‐ and high‐order feature interaction and was successfully used in Click Through Rate (CTR) prediction^39^ and MDA prediction.^36^ To further enhance the prediction performance, we present an improved DeepFM model, MRDPDA, which is regularized separately by each of multiple Laplacian instead of one Laplacian from simple superposition of multiple similarities about piRNAs and diseases.

The proposed MRDPDA includes three parts, which are embedding layer, FM and MLP components. FM and MLP components share the same inputs from the embedding layer and final output is calculated according to the outputs of FM and MLP:

(11) $$\hat{y}=\text{sigmoid}\left(y_{\mathrm{FM}}+y_{\mathrm{MLP}}\right)$$

**FIGURE 2:**
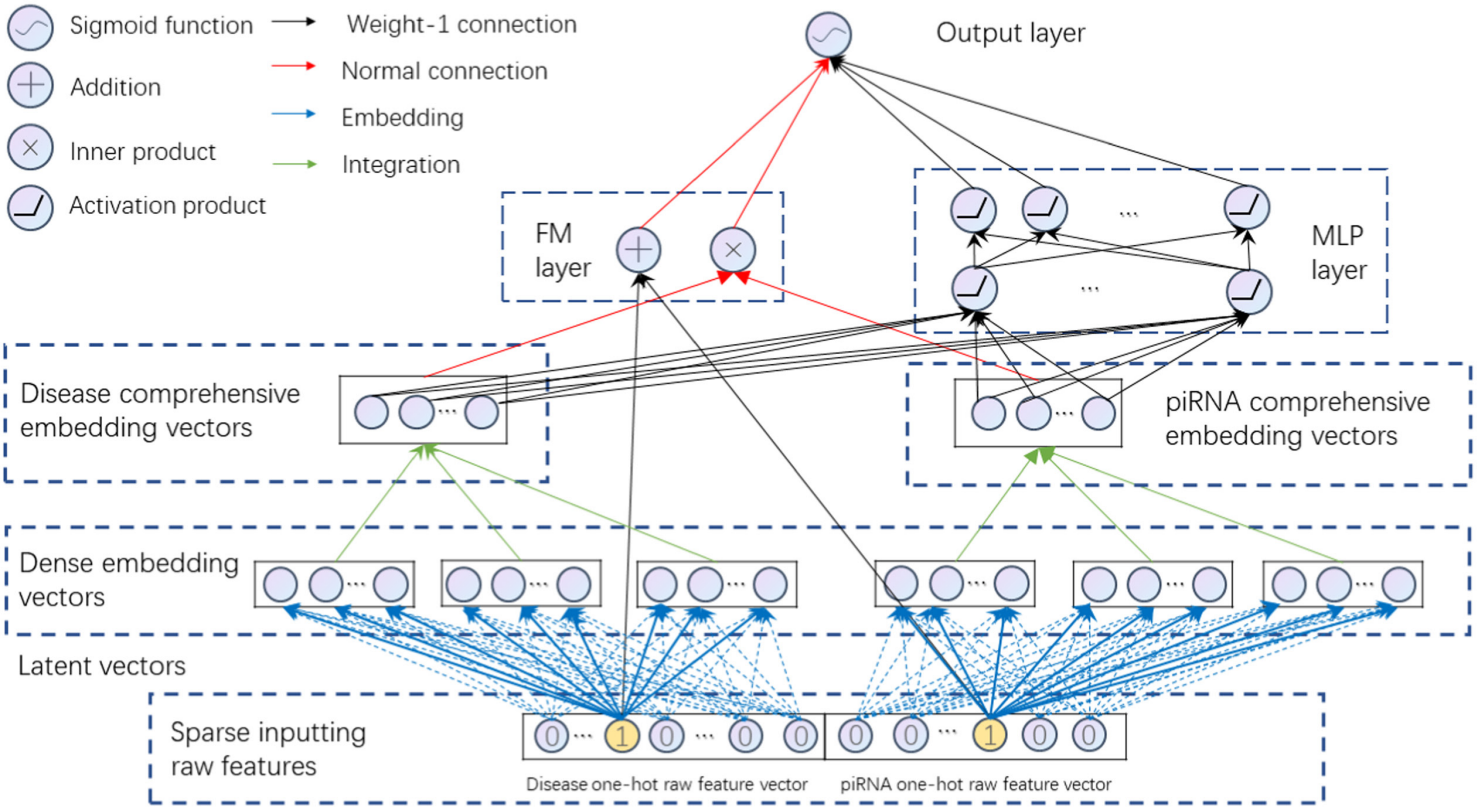
The framework of the DeepFM model on PDA prediction.

The proposed framework is shown in Figure 2 and details are described in the sequel.

#### 2.4.1 Embedding layer

**FIGURE 3:**
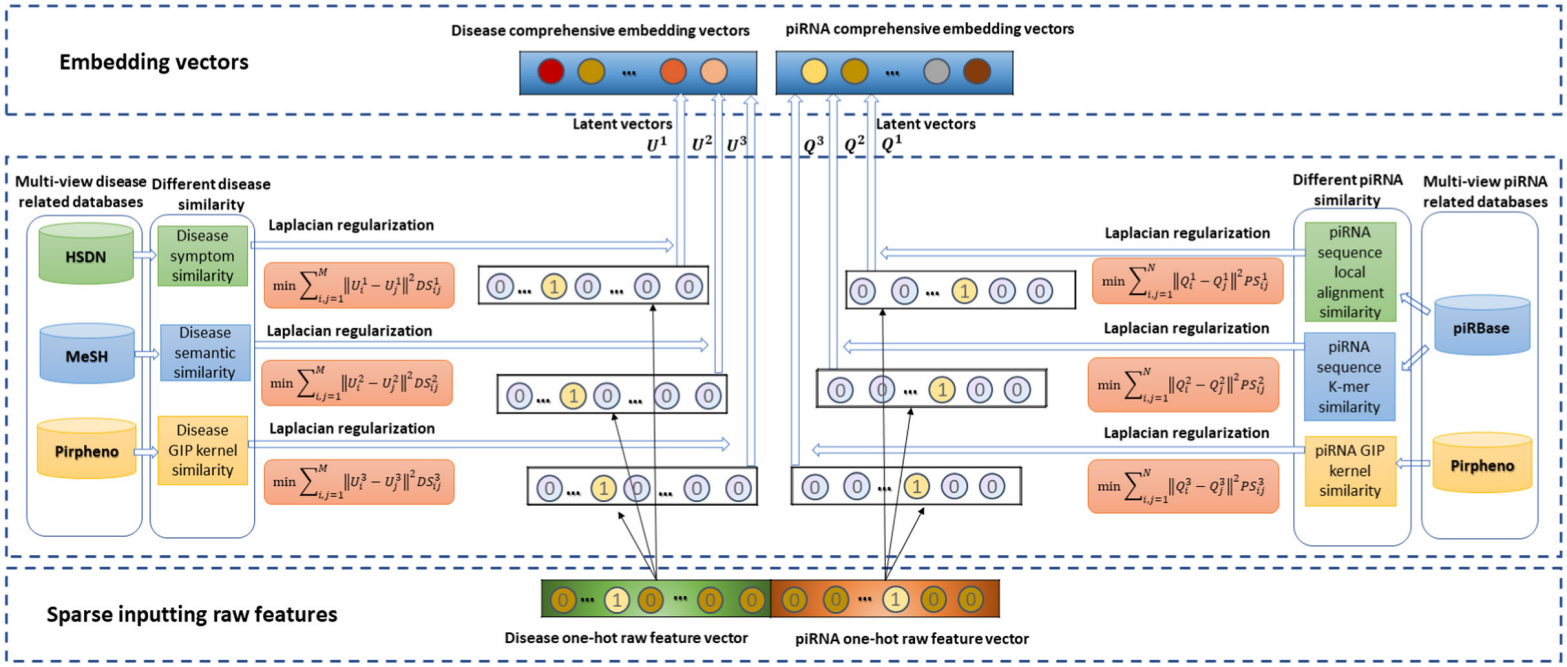
The structure of the embedding layer about similarity‐level data fusion.

Ding et al.^36^ demonstrate the embedding layer plays an important role in DeepFM, so we aim to improve the embedding layer by Laplacian regularization of separate similarities. The structure of the embedding layer about similarity‐level data fusion is shown in Figure 3.

Three types of piRNA similarities (piRNAs k‐mer, piRNA local alignment and GIP kernel) and three types of disease similarities (diseases semantics, diseases symptom and GIP kernel) are taken into consideration in this study. One‐hot encoding representations of a piRNA and a disease are utilized in the proposed model as inputs. Six Laplacian regularization of separate similarities are applied and Laplacian eigenmaps are used to initialize the weights for the embedding layer. As described previously, ${\mathrm{PS}}_{\mathrm{ij}}^{1}$, ${\mathrm{PS}}_{\mathrm{ij}}^{2}$ and ${\mathrm{PS}}_{\mathrm{ij}}^{3}$ are local alignment, k‐mer and GIP kernel similarities of the i‐th and j‐th piRNAs, respectively. $Q_{i}^{1}$ and $Q_{j}^{1}$ are the column latent vectors of the i‐th and j‐th piRNAs about the piRNA sequence local alignment similarity. The Laplacian regularization for this similarity can be computed as a minimum $R^{1}$ optimization problem as follows:

$$R^{1}=\frac{1}{2}\sum_{i,j=1}^{N}{\left\|Q_{i}^{1}-Q_{j}^{1}\right\|}^{2}{\mathrm{PS}}_{\mathrm{ij}}^{1}$$

$$=\sum_{i=1}^{N}{\left(Q_{i}^{1}\right)}^{T}Q_{i}^{1}D_{\mathrm{ii}}^{p}-2\sum_{i,j=1}^{N}{\left(Q_{i}^{1}\right)}^{T}Q_{j}^{1}{\mathrm{PS}}_{\mathrm{ij}}^{1}$$

(12) $$=\mathrm{Tr}\left(Q^{1}D^{p}{\left(Q^{1}\right)}^{T}\right)-\mathrm{Tr}\left(Q^{1}{\mathrm{PS}}^{1}{\left(Q^{1}\right)}^{T}\right)=\mathrm{Tr}\left(Q^{1}L_{p}^{1}{\left(Q^{1}\right)}^{T}\right)$$

where $D^{p}$ is a diagonal matrix with $D_{\mathrm{ii}}^{p}=\sum_{j=1}^{N}{\mathrm{PS}}_{\mathrm{ij}}^{1}$, 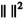 is the square of 2 norm, Tr is the matrix trace, $Q^{1}$ is the latent feature matrix whose i th column is the corresponding latent vectors of the i‐th piRNA and $L_{p}^{1}=D^{p}-{\mathrm{PS}}^{1}$ is the Laplacian matrix.

In the same way, the Laplacian regularization for the k‐th piRNA similarity and the t‐th disease similarity can be represented as minimum $R^{k}$ and $R^{t}$ optimization problem as follows:

(13) $$R^{k}=\frac{1}{2}\sum_{i,j=1}^{N}{\left\|Q_{i}^{k}-Q_{j}^{k}\right\|}^{2}{\mathrm{PS}}_{\mathrm{ij}}^{k}=\mathrm{Tr}\left(Q^{k}L_{p}^{k}{\left(Q^{k}\right)}^{T}\right)\;\left(k=1,2,3\right)$$

(14) $$R^{t}=\frac{1}{2}\sum_{i,j=1}^{M}{\left\|U_{i}^{t}-U_{j}^{t}\right\|}^{2}{\mathrm{DS}}_{\mathrm{ij}}^{t}=\mathrm{Tr}\left(U^{t}L_{d}^{t}{\left(U^{t}\right)}^{T}\right)\;\left(t=1,2,3\right)$$

Where $Q_{i}^{k}$ and $Q_{j}^{k}$ are the column latent vectors of the i‐th and j‐th piRNA about the k‐th piRNA similarity,$\;U_{i}^{t}$ and $U_{j}^{t}$ are the column latent vector of the i‐th and j‐th disease about the t‐th disease similarity, $Q^{k}$ is the latent feature matrix whose i‐th column is the corresponding latent vectors of the i‐th piRNA, $U^{t}$ is that of the t‐th disease, $L_{p}^{k}$ and $L_{d}^{t}$ are the Laplacian matrixes.

Suppose the loss function of MRDPDA is $J\left(Q^{k},U^{t},O\right)$, where $Q^{k}$ and $U^{t}$ are the latent feature matrices of piRNAs and diseases in the embedding layer, respectively.O is the set of all the rest parameters and J is the cross‐entropy loss function. We propose a unified and extended objective function $F\left(Q^{k},U^{t},O\right)$, which combines the cross‐entropy loss and Laplacian regularizations as follows:

$$\min F\left(Q^{k},U^{t},O\right)=\min J\left(Q^{k},U^{t},O\right)$$

$$+\sum_{k=1}^{3}{p_{\lambda }}_{k}\mathrm{Tr}\left(Q^{k}L_{p}^{k}{\left(Q^{k}\right)}^{T}\right)$$

(15) $$+\sum_{t=1}^{3}{d_{\lambda }}_{t}\mathrm{Tr}\left(U^{t}L_{d}^{t}{\left(U^{t}\right)}^{T}\right)$$

where ${p_{\lambda }}_{k}$and ${d_{\lambda }}_{t}$ are the regularization parameters that are used to balance the MRDPDA loss term and regularization term.

It is still noteworthy that sparse disease similarity matrix from MeSH cause the failure of Laplacian regularization. If a disease has no similarities information, a small perturbation of 0.001 is set in the column and row of this disease to ensure the regularization operation.

Different view similarity values can reflect the associations from different criteria and latent vectors by individual regulation of each similarity are set for the associations between the nodes' weights in the different fields of embedding layer. The comprehensive embedding vectors about piRNA and disease are integrated by the average of the obtained embedding vectors.

Following the precious study practices,^36^ low dimensional feature representations from the Laplacian eigenmaps of each node is used for initializing node's corresponding weights in the dense embedding layer.

#### 2.4.2 FM and MLP components

A standard FM and MLP components are used in our model and it is responsible for the interaction between low‐order features.

As shown in Figure 2, the output of FM component is the sum of the addition unit and the inner product units. The addition unit reflects the linear interactions among features and the inner product represents the effect of order‐2 feature interactions. The output of FM is as follows:

(16) $$y_{\mathrm{FM}}=\left\langle w,x\right\rangle +\sum_{j_{1}=1}^{d}\sum_{j_{2}=j_{1}+1}^{d}\left\langle V_{i},V_{j}\right\rangle x_{j_{1}}x_{j_{2}}$$

Where $\;x=\left[x_{\text{Field}\_\text{piRNA},}\;x_{\text{Field}\_\text{disease}}\right]$ is the d‐dimensional vector which contains one‐hot raw feature vectors of a piRNA and a disease, $w\in R^{d}$ is network parameters to measure impact of order‐1 features' interaction, $V_{i}$ and $V_{j}$ is the corresponding latent vectors of raw features $x_{i}$ and $x_{j}$, respectively.

MLP component is a feed‐forward multi‐layer neural network and used to learn high‐order feature interactions according to the embedding vectors. The comprehensive embedding vectors about piRNA and disease are denoted as $e_{p}$ and $e_{d}$, respectively. The input of MLP component $x^{\left(0\right)}$, the forward process $x^{\left(l+1\right)}$ and the output of MLP $y_{\mathrm{MLP}}$ are as follows:

(17) $$x^{\left(0\right)}=\left[e_{p},e_{d}\right]$$

(18) $$x^{\left(l+1\right)}=\sigma \left(W^{\left(l\right)}x^{\left(l\right)}+b^{\left(l\right)}\right)\;l=0,\ldots ,H$$

(19) $$y_{\mathrm{MLP}}=\sigma \left(W^{\left(H\right)}x^{\left(H\right)}+b^{\left(H\right)}\right)$$

Where σ is the active function, $W^{\left(l\right)}$ and $b^{\left(l\right)}$ are the weights and bias at the l‐th layer, $W^{\left(H\right)}$and $b^{\left(H\right)}$ are that at the H hidden layers in MLP.

## 3 RESULTS

### 3.1 Evaluation methods and hyper‐parameters

In our study, five‐fold cross‐validation and independent test are employed to evaluate the performance of models. All experiments are run on a computer with an NVIDIA GeForce RTX 4070 graphics card, i7‐10700KF CPU, 32GB of RAM and 4.5 TB of storage. The area under the receiver operating characteristics curve (AUC) and under the precision recall curve (AUPR) are used as performance metrics. The higher the AUC and AUPR values, the better the predictive performance of the model is.

**FIGURE 4:**
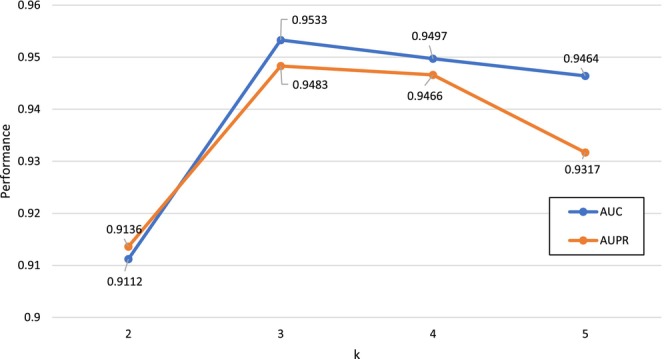
The predictive results of various ranges of *k*.

Many hyper‐parameters influence the prediction performance. These parameters were empirically selected or chose based on performance evaluation in this study. In the step of similarity calculation, the hyper‐parameter k denotes the length of k‐mer fragment. If the choice of k value is too small, *k*‐mer fragments will not fully reflect the characteristics of the piRNA sample and affect the prediction performance. If the choice of k value is too large, it will bring the large computational overhead. The predictive results with different number of k are illustrated in Figure 4. Taking into account both prediction performance and computational efficiency, the k value is empirically set to 3.

In the step of reliable negative samples selection, autoencoder model with a latent size of 32 are used. The hidden layer dimensions are 256, 64, 32, 64, 256 and input and output dimensions are 564. Mini‐batch gradient descent with the batch size of 64 and Adam optimizer are applied. In prediction model construction part, the embedding size embedding_size is the first key hyper‐parameter, which is searched from $\left\{5,10,15,\ldots ,55,60\right\}$. The results on five‐fold CV show that the optimal *embedding_size* value is 20. The regularization coefficients ${p_{\lambda }}_{k}$ and ${d_{\lambda }}_{t}$ are set as 0.01. The hidden layer dimensions of MLP part are 64 and 32, the activation function is ReLU. The learning rate is 0.001, the batch size is 64 and the dropout rate was set to 0.5.

### 3.2 Effectiveness of the improvements

MLRDFM is an improved deepFM model which has successfully been used in MDA prediction.^36^ MRDPDA improves MLRDFM performance on PDA by structural modification of the embedding layer and selection of reliable negative set. To illustrate the effectiveness of these two improvements, we respectively compare the proposed method with MLRDFM with random unlabeled samples and reliable negative samples. Five‐fold cross‐validation experiments are conducted and the benchmark dataset proposed in this paper are used.

**FIGURE 5:**
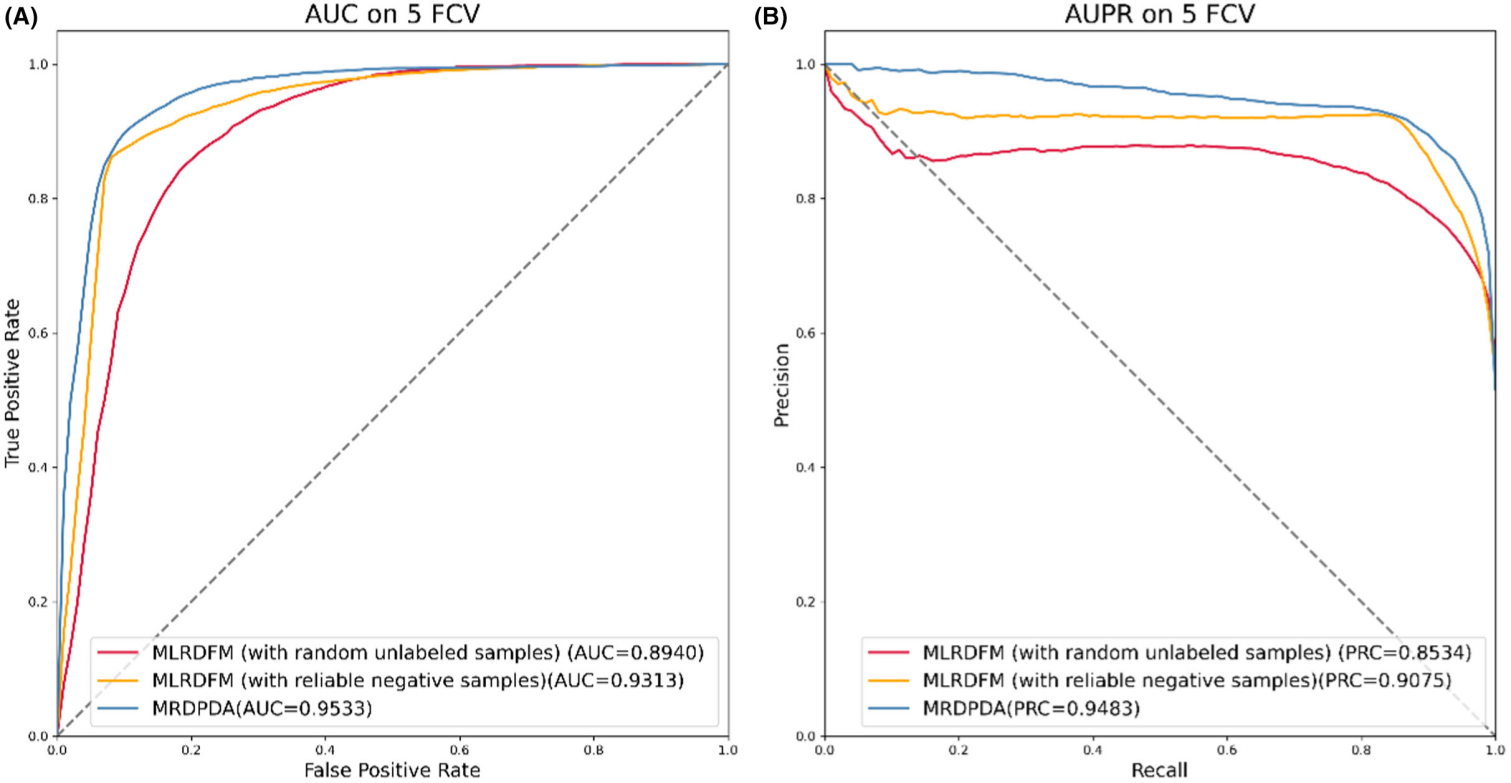
The comparison of AUC and AUPR of the three models.

The AUC and AUPR of those three models' prediction results are shown in Figure 5, from which we can see the following: (1) Compared with the orange curves (MLRDFM with random unlabeled samples), the red curves (MLRDFM with reliable negative samples) achieve the better performance, indicating that selection of reliable negative set by learning from positive samples contributes to the progress; (2) Compared with the simply superimposition and random initialization, six Laplacian regularization of separate similarities and initialization of Laplacian eigenmaps in the embedding layer help increased performance; (3) Overall MRDPDA yielded the best identification performance with the AUC of 0.9533 and AUPR of 0.9483.

### 3.3 Performance comparison with existing methods

Various PDA methods have been proposed and the three PDA methods with public code (iPiDA‐GCN,^24^ GAPDA‐LGAT^23^ and ETGPDA^26^) are chosen to compare. iPiDA‐GCN is a PDA predictor based on graph convolutional networks (GCN) for learning hidden association patterns in different biological networks. GAPDA‐LGAT is a PDA method based online graph attention networks by extending heterogeneous networks to replace dichotomous networks. ETGPDA is a method based on GCN with the embedding transformation, which coverts piRNA and disease embeddings into the same space.

First, five‐fold cross validation (5‐CV) test on the benchmark dataset proposed in our paper are used and computation time of them are measured respectively. Furthermore, the independent test dataset was downloaded from Hou et al.^24^ for performance comparison. Table 2 demonstrates the results of performance comparison.

**TABLE 2:** The performance comparison of the four methods.

	Methods	MRDPDA	iPiDA‐GCN	ETGPDA	GAPDA‐LGAT
5‐CV	AUC (%)	**95**.**33**	88.07	90.53	87.38
	AUPR (%)	**94.83**	83.81	88.66	81.75
	Run time (ms)	**141,202**	343,612	142,418	289,607
Independent test	AUC (%)	**71**.**76**	71.49	70.58	56.12
	AUPR (%)	69.01	**70**.**36**	68.93	55.37

*Note*: Significance for bold values is the best performance.

From the 5‐CV results, it can be seen that MRDPDA achieves the highest AUC (95.33%) and AUPR (94.83%). Additionally, our method takes141,202 ms, which is the fastest of four methods. It is slightly faster than ETGPDA (142,418 ms), is approximately twofold faster than GAPDA‐LGAT (289,607 ms) and iPiDA‐GCN (343,612 ms). This reveals our method is more effective and faster than the other three methods.

From independent test results, AUC of MRDPDA is still the highest and its AUPR is slightly lower than that of iPiDA‐GCN. It is worth noting that GAPDA‐LGAT is constructed for limited dataset and only predict the piRNAs associated with 13 diseases in the independent test dataset, so the prediction result is evaluated by these associations.

### 3.4 Case studies

To evaluate the ability of MRDPDA for predicting associated piRNAs for new diseases, case studies of two important diseases ‘Alzheimer's disease’ and ‘Gastric cancer’ were reported. As in iPiDA‐sHN,^30^ we remove the pairs related to Alzheimer's disease and re‐trained the model with the remaining samples. The trained model is used to identify the missing known associations of Alzheimer's disease. The top 10 piRNA associated with these two diseases are listed in Table 3. Six identified piRNAs associated with ‘Alzheimer's disease’ and eight identified associations of ‘Gastric cancer’ have been validated by literature mining. For examples, by high throughput technologies, the upregulation of piR‐hsa‐22564 and piR‐hsa‐14621, the downregulation of piR‐hsa‐14962 may be correlated with the increased risk of Alzheimer's disease.^40^ piR‐hsa‐1282 dysregulation associated with gastric cancer was validated by RT‐PCR and the animal model experiments in vivo.^41^ These prediction results demonstrate the effectiveness of the proposed method. The unverified piRNA‐disease relationships can be considered as candidates and investigated in the further research.

**TABLE 3:** The top 10 piRNAs associated Alzheimer's disease (AD) and Gastric cancer (GC) predicted by our study.

Rank	piRNAs	AD	Rank	piRNAs	GC
1	piR‐hsa‐22564	Yes	1	piR‐hsa‐1282	Yes
2	piR‐hsa‐14621	Yes	2	piR‐hsa‐1963	Yes
3	piR‐hsa‐14962	Yes	3	piR‐hsa‐7193	Yes
4	piR‐hsa‐1251	No	4	piR‐hsa‐27492	Yes
5	piR‐hsa‐25074	Yes	5	piR‐hsa‐5370	Yes
6	piR‐hsa‐28771	Yes	6	piR‐hsa‐26441	Yes
7	piR‐hsa‐4685	Yes	7	piR‐hsa‐20266	Yes
8	piR‐hsa‐5967	No	8	piR‐hsa‐17444	Yes
9	piR‐hsa‐26359	No	9	piR‐hsa‐27007	No
10	piR‐hsa‐32046	No	10	piR‐hsa‐15404	No

## 4 DISCUSS AND FUTURE DIRECTIONS

Various association prediction tasks are common in bioinformatics studies, such as ligand‐receptor interactions, drug‐target interactions, biomolecule‐disease associations and so on.^42^, ^43^, ^44^ Compared with the available MDA, CDA and LDA computational tools, PDA research is still in its initial phase.^45^ Challenges remain in representation learning capacity, the quality and quantity of the benchmark set. In our study, we have developed a novel method, MRDPDA, for predicting PDAs based on limited data from multiple sources. This method effectively integrates a deepFM model with regularizations of several separate Laplacians calculated from multiple yet limited datasets, instead of simply calculating an average similarity network to regularize the deepFM.

Moreover, a unified objective function is proposed to combine embedding loss about similarities to ensure that the embedding is suitable for the prediction task. In addition, we have constructed a relatively balanced benchmark from piRPheno with a deep autoencoder for selecting reliable negative samples. This method has achieved the best performance on 5‐CV and independent test among latest computing methods. Case studies have further demonstrated the effectiveness. Clearly, there is still some room for improvement in the future:

### 4.1 Data perspective

The quality and quantity of current PDA datasets still need to be improved. Because of the limited number and strong inclusion relations of diseases, we would try to design rationally merged method and build PDA data management platform. In addition, we would try to train a unified model based on three or more datasets in the setting of meta learning.

### 4.2 Algorithm perspective

Future PDA algorithm research can be considered from two ways. On the one hand, we would try to develop the new prediction model which is suitable for highly imbalanced PDA data. It is still noteworthy that PDA databases have highly abundant one‐to‐one associations. 88.62% and 86.13% piRNAs are associated with only one disease in piRDisease and MNDR, respectively. On the other hand, we will consider various learning strategies to improve the PDA prediction performance. For example, boosting models have obtained better performance in prediction of ligand‐receptor interactions^46^, ^47^, ^48^ by combining multiple weak learners. Once the PDA data is sufficient, ensemble learning strategy will be applied.

## AUTHOR CONTRIBUTIONS

**Yajun Liu:** Conceptualization (equal); formal analysis (equal); funding acquisition (equal); investigation (equal); methodology (equal); validation (equal); visualization (equal); writing – original draft (equal). **Fan Zhang:** Data curation (equal); visualization (equal). **Yulian Ding:** Investigation (equal); methodology (equal). **Rong Fei:** Investigation (equal); visualization (equal). **Junhuai Li:** Investigation (equal). **Fang‐Xiang Wu:** Conceptualization (equal); funding acquisition (lead); methodology (equal); project administration (lead); supervision (lead).

## FUNDING INFORMATION

This work was supported by the Young Scientists Fund of the National Natural Science Foundation of China (Grant No. 62202374 and U22A2041), the Natural Science Basic Research Program of Shaanxi Province of China (Program No. 2024JC‐YBMS‐484), the China Postdoctoral Science Foundation (2021 M693887) and Natural Science and Engineering Research Council of Canada (NSERC).

## CONFLICT OF INTEREST STATEMENT

The authors declare no potential conflict of interests.

## Data Availability

The code and dataset of MLRDFM are freely available at https://github.com/liuyajun1007/PDA.
